# Adverse Socioeconomic Conditions and Oocyst-Related Factors Are Associated with Congenital Toxoplasmosis in a Population-Based Study in Minas Gerais, Brazil

**DOI:** 10.1371/journal.pone.0088588

**Published:** 2014-02-11

**Authors:** Ericka Viana Machado Carellos, Gláucia Manzan Queiroz de Andrade, Daniel Vitor Vasconcelos-Santos, José Nélio Januário, Roberta Maia Castro Romanelli, Mery Natali Silva Abreu, Fabiana Maria da Silva, Ivy Rosa Coelho Loures, Juliana Queiroz de Andrade, Waleska Teixeira Caiaffa, UFMG Congenital Toxoplasmosis Brazilian Group (UFMG CTBG)

**Affiliations:** 1 Department of Pediatrics, School of Medicine, Universidade Federal de Minas Gerais, Belo Horizonte, Minas Gerais, Brazil; 2 Department of Ophthalmology, School of Medicine, Universidade Federal de Minas Gerais, Belo Horizonte, Minas Gerais, Brazil; 3 Center for Newborn Screening and Genetic Diagnosis, School of Medicine, Universidade Federal de Minas Gerais, Belo Horizonte, Minas Gerais, Brazil; 4 School of Nursing, Universidade Federal de Minas Gerais, Belo Horizonte, Minas Gerais, Brazil; 5 Hospital Infantil João Paulo II, Fundação Hospitalar do Estado de Minas Gerais, Belo Horizonte, Minas Gerais, Brazil; 6 School of Medicine, Universidade Federal de Minas Gerais, Belo Horizonte, Minas Gerais, Brazil; 7 Department of Preventive and Social Medicine, Observatory of Urban Health, School of Medicine, Universidade Federal de Minas Gerais, Belo Horizonte, Minas Gerais, Brazil; Institut national de la santé et de la recherche médicale - Institut Cochin, France

## Abstract

**Objective:**

Congenital toxoplasmosis is a public health problem in Brazil. This study aimed to determine risk factors associated with congenital toxoplasmosis in Minas Gerais which is the second largest Brazilian State based on number of inhabitants, and its territorial extension is larger than that of France. Methods: Population-based case-control study to assess the association between congenital toxoplasmosis and maternal exposure to infection risk factors. The study included mothers/children participating in the Minas Gerais Newborn Screening Program. The cases consisted of 175 mothers of infected children, and the controls consisted of 278 mothers of children without suspected infection. The associations were assessed through binomial logistic regression with *p*≤0.05.

**Results:**

The variables associated with lower probability of toxoplasmosis were: older mother age (OR = 0.89; CI95% = 0.85–0.93), higher level of education (OR = 0.85; CI95% = 0.78–0.92), access to potable water (OR = 0.21; CI95% = 0.08–0.51), and home with flush toilet (OR = 0.18; CI95% = 0.04–078). The variables associated with higher probability of infection were: cats in the neighborhood (OR = 2.27; CI95% = 1.27–4.06), owning or visiting homes with domestic cats (OR = 1.90; CI95% = 1.09–3.31), handling the soil (OR = 2.29; CI95% = 1.32–3.96), and eating fresh meat not previously frozen (OR = 3.97; CI95% = 2.17–7.25). After stratification according region of residence (rural or urban/peri-urban), home with flush toilet and consumption of treated water were protective against the disease only in the rural stratum.

**Conclusions:**

In Minas Gerais, congenital toxoplasmosis has been associated with poor socioeconomic conditions. Considering maternal exposure to sources of *Toxoplasma gondii*, the predominating risk factors were those related to the ingestion of oocysts. It is expected that these results will contribute to development of a program for prevention of congenital toxoplasmosis adapted to the reality of the population of Minas Gerais. The differences between populations living in rural and urban areas regarding the main risk factors for toxoplasmosis point to the need of considering regional specificities in planning strategies to control congenital toxoplasmosis.

## Introduction

Toxoplasmosis is a disease caused by the ubiquitous protozoan *Toxoplasma gondii*. Its prevalence varies according to geographic region, as well as socioeconomic and cultural factors [1].

The main social impact of toxoplasmosis in humans is associated with vertical infection. In this situation, the parasite is capable of causing severe disease, with short and/or long term sequelae. Infected newborns may be asymptomatic at birth or display a wide array of signs/symptoms, from unspecific systemic involvement to severe neurological and ocular damage, as well as hearing impairment [2].

There are three major strategies to control congenital toxoplasmosis: prenatal and newborn screening and, health education. The educational approach is the only measure truly capable of preventing infection among pregnant women. However, educational measures will have an impact on the women's behavior only if they are adequately sensitized to the need of changing habits [3], [4].

As the sources of infection are multiple and vary across regions [5], [6], it is important to identify the most relevant epidemiological factors in order to adjust the prophylactic instructions to the context of the target population. In Brazil, especially in the State of Minas Gerais, there are few studies with adequate design to evaluate the risk factors involved with the regional transmission of toxoplasmosis. Thus, a multidisciplinary research group has been assembled to measure the impact of congenital toxoplasmosis on the population of the State of Minas Gerais.

Congenital toxoplasmosis was screened in the State using dried blood collected from the participants of the Newborn Screening Program of the State of Minas Gerais (PETN-MG) over a period of seven months. The screening pointed to high prevalence of the infection, 13 cases in every 10 000 newborns, and a regional social inequity regarding prevalence rates of the infection [7].

Given the importance of toxoplasmosis infection in this Brazilian context this study aims at identifying the main risk factors associated with congenital toxoplasmosis in the State of Minas Gerais and contributing to developing a prevention program adapted to the regional context.

## Patients and Methods

### Ethics statement

The study protocol followed the tenets of the Declaration of Helsinki. The study received approval in all required instances: Universidade Federal de Minas Gerais (UFMG), School of Medicine, Departments of Pediatrics (n^o^. 69/2007); Centro de Treinamento e Referência em Doenças Infecciosas Orestes Diniz (n^o^. 16/2007), and UFMG Ethics Committee (ETIC 510/07). Written informed consent was obtained from the parents or guardians of all participating children.

### Study design

A case-control approach was used to assess the association between congenital toxoplasmosis and the exposure of pregnant women to risk factors.

### Scenario and population

The study was conducted in Minas Gerais, a state in Southeastern Brazil with 853 municipalities and 19,597,330 inhabitants in total, spread over an area of 586,520 km^2^ according to the last census by the Brazilian Institute of Geography and Statistics (IBGE, 2010). The study included newborns participating in the PETN-MG.

### Diagnosis of congenital toxoplasmosis

**Figure 1:**
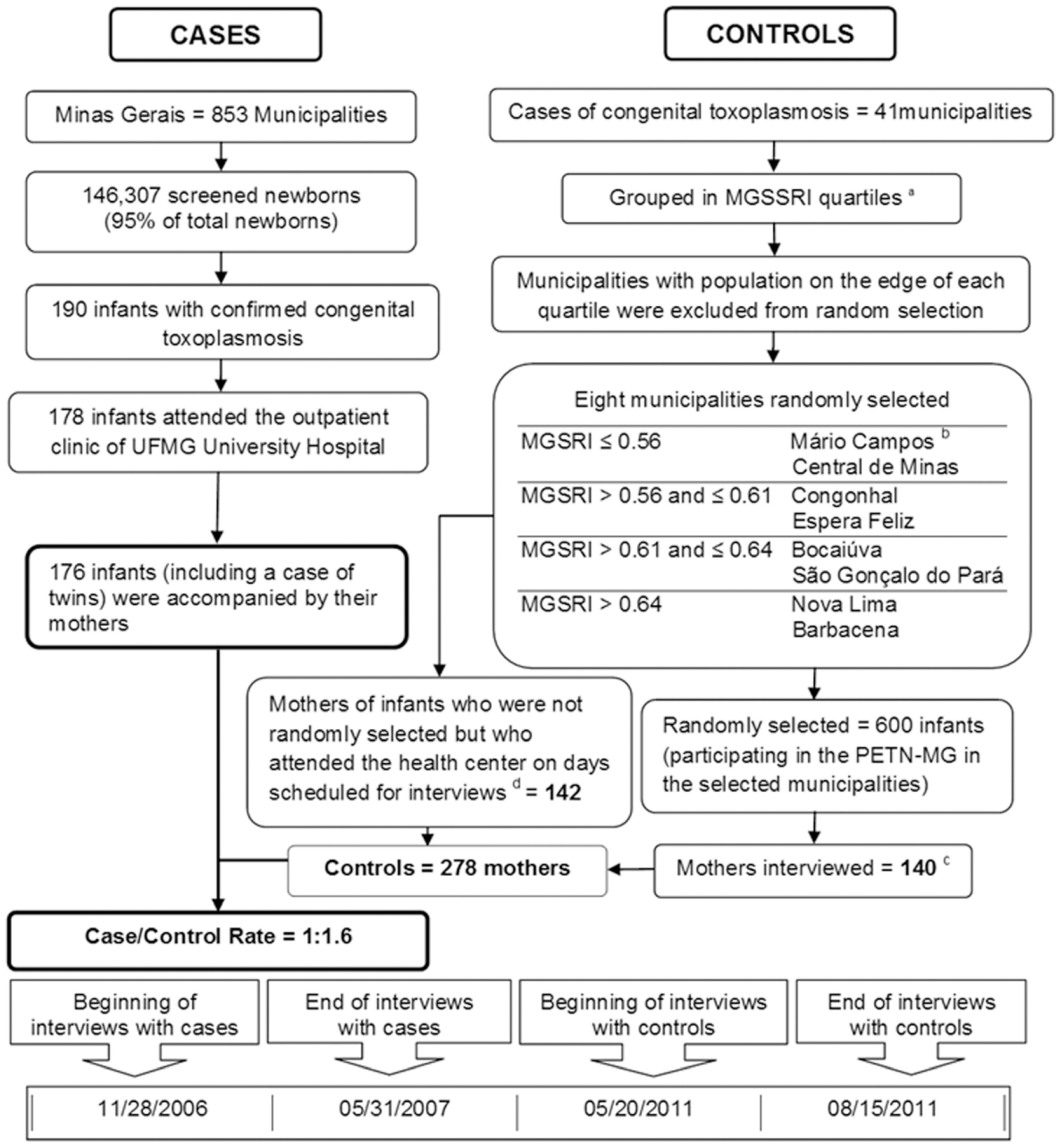
Flow chart of the case-control study for assessment of risk factors for congenital toxoplasmosis in the State of Minas Gerais. ^a^ Minas Gerais State Social Responsibility Index. ^b^ Municipality from the same stratum that replaced Joaíma due to unsuccessful contact. ^c^ Four mothers/children were excluded due to suspected gestational/congenital toxoplasmosis. ^d^ Included mothers of children aged up to six months. In Barbacena, interviews took place at the central vaccination center of the municipality.

The study included as cases the mothers of newborns confirmed with congenital toxoplasmosis (Figure 1) according to the screening carried out among PETN-MG participants from November 2006 through May 2007. In summary, newborn screening (covering 95% of the newborns in the State) was based on anti-*T.gondii* IgM tests of dried blood collected in filter paper from newborns. Confirmative plasma serum tests were run on cases with positive or undetermined results – anti-*T.gondii* IgG and IgM antibodies (children/mothers), anti-*T. gondii* IgA antibodies (children). The infants were referred to the outpatient clinic of the UFMG University Hospital for clinical examination and complementary investigations. All of them received treatment as soon as the diagnosis was confirmed [7].

The criteria for confirmed congenital toxoplasmosis were: (1) positive anti-*T.gondii* IgM and/or IgA and positive IgG until age of 6 months; (2) negative anti-*T.gondii* IgM/IgA and positive IgG associated with retinochoroidal lesions within the first six months of life; (3) persistence of positive anti-*T.gondii* IgG results until age of 12 months [8].

### Selection of controls

Once the number of cases had been defined (n = 175), the number of controls was estimated with reference to the 5% level of significance (α), and the test power (1 − β) of 80%, based on reports in the literature.

The population-based controls were selected among mothers of infants included in the aforementioned program in the year 2011. These newborns were not tested for toxoplasmosis, as the program screening had not included this disease in the period under scrutiny. From 141 out of 853 municipalities in the State that reported cases of congenital toxoplasmosis from November 2006 through May 2007, four strata were creating according to performance in the Minas Gerais State Social Responsibility Index, a socioeconomic indicator created by the Center for Public Policy Studies at Fundação João Pinheiro with a view to depicting the level of development of the municipalities in the State [7]. Each stratum included two municipalities, and the number of mothers/children randomly selected per municipality was proportional to the number of inhabitants (Figure 1). In total, eight municipalities were randomly selected among the 141 municipalities.

### Inclusion and exclusion criteria for cases and controls

Mothers of infants with confirmed congenital toxoplasmosis who brought their children to outpatient clinic of HC-UFMG and could be interviewed were included as cases.

The controls included a randomly selected sample of mothers/children previously invited to participate who went to the health center in the assigned municipalities. The mothers of infants randomly selected who missed their interviews were replaced by mothers of children aged up to seven months who went to the health center for regular examination, immunization or collection of dried blood with filter paper within the scope of PETN-MG. Since these newborns were not tested for toxoplasmosis, as the program screening had not included this disease in the period under scrutiny, a careful investigation was performed in order to exclude mothers/children suspected of acute gestational toxoplasmosis/congenital toxoplasmosis, through mother inquiry, prenatal appointments and children health reports.

### Data collection

Interviews with the cases were carried out from November 2006 through May 2007, and with the controls from May through August 2011 (Figure 1). Upon informed consent, the mothers were interviewed using a semi-structured questionnaire with questions on socioeconomic and demographic aspects, residence region, prenatal appointments, pets, behavior and dietary habits during pregnancy, and previous knowledge about ways of preventing toxoplasmosis. The researchers interviewed the mothers when their children had the first appointment at the HC-UFMG.

The mothers of the controls were invited for interviews at the main health center of their home municipalities. All data collection procedures were standardized. Pediatricians in training participated in all phases of data collection under the supervision of the authors of this study.

### Statistical analysis

A binomial logistic regression model was used to assess the association of every risk factor with cases and controls in two steps. In the first, cases and controls were compared in a univariate analysis, followed by a forward multivariate analysis using every variable with *p*<0.25 at a time. The remaining final model included variables with *p*≤0.05 and those with epidemiological criteria after assessment of collinearity [9].

In the second step, the cases/controls were stratified in two groups according to region of residence (urban or rural). The multivariate analysis was carried out once again, this time considering only the variables that remained in the final model as statistically significant. For every step, the fit of the multiple regression model was assessed using Hosmer & Lemeshow's statistics [9].

The association measure was the odds ratio, with confidence interval of 95% and significance level of 5% [10]. The software package SPSS 15.0 was used for the analyses.

## Results

### The sample

Congenital toxoplasmosis was confirmed at the age of 12 months for a total of 190 out of 146 307 screened newborns from November 2006 through May 2007. The referral outpatient clinic provided health care to 178 of these children (including one case of twins), and 12 children were followed at distance in their own municipality of residence. Only two pairs of mother/child refused to participate and therefore were excluded from the analysis. Thus, 175 mothers of infected children participated in the epidemiological interview (Figure 1).

The group of controls comprised 282 mothers interviewed in the eight randomly selected municipalities. Four mother/child pairs were excluded: one because of suspected maternal toxoplasmosis and anti-*Toxoplasma* treatment during pregnancy, and three because of reports of symptoms compatible with congenital toxoplasmosis. The final sample of 278 control mothers resulted in a case/control ratio of 1∶1.6 (Figure 1), with detection power ranging from 65 to 100%. These figures consider the lowest and the highest difference found between cases and controls in relation to exposure to risk factors: from 9.7% (reports of cockroaches at home) to 29.5% (reports of visiting places with cats).

Considering the control group, no statistical difference was found between mothers of infants randomly selected and the mothers who replaced those who missed their interviews, concerning their demographic and socioeconomic characteristics, life habits, and environmental risk factors.

As expected by study design, municipalities of the cases and the controls showed no statistical difference concerning the MGSRI indicator (*p* = 0.51) and number of inhabitants (*p* = 0.94).

The analysis of the children's age showed that the group of cases had lower median age than the group of controls – 58 and 94 days respectively (*p*<0.01). However, no statistical difference was found in relation to the risk factors reported by the mothers of the control group after categorizing the group in quartiles of the children's age.

### Risk factors associated with toxoplasmosis in the State of Minas Gerais

The comparative univariate analysis pointed to association of congenital toxoplasmosis with mothers' lower educational level, lower income, higher percentage of adolescent mothers, rural residence, and reports of precarious living conditions (Table 1).

**Table 1:** Univariate analysis of demographic and socioeconomic characteristics of mothers of children with congenital toxoplasmosis and their controls identified in the scope of the newborn screening program in the State of Minas Gerais.

Variables	Cases (n = 175)			Controls (n = 278)			*p*- value	OR (CI 95%)
	n	Frequency(%)	Median (P25;P75)	n	Frequency (%)	Median (P25;P75)		
Mother age at delivery	175	-	22 (19; 28)	276	-	27 (22; 32)	<0.01	0.91 (0.88–0.94)
Adolescent mother (up to 19 years old)	175	55 (31.4)	-	276	33 (12)	-	<0.01	3.37 (2.08–5.47)
Mother's years of at school a	173	-	7 (5; 10)	278	-	11 (8; 11)	<0.01	0.79 (0.74–0.85)
Income lower than 1 minimum salary b	168	44 (26.2)	-	276	27 (9.8)	-	<0.01	3.27 (1.93–5.53)
Domicile in a rural area	174	58 (33.3)	-	278	20 (7.2)	-	<0.01	6.45 (3.71–11.2)
Own home (legalized)	173	91 (52.6)	-	278	167 (60.1)	-	0.12	0.74 (0.50–1.08)
Masonry house c	175	164 (93.7)	-	275	273 (99.3)	-	<0.01	0.11 (0.02–0.50)
Number of rooms	173	-	3 (2; 4)	278	-	3 (3; 4)	0.68	0.95 (0.82–1.1)
Number of dwellers	174	-	3 (2; 5)	278	-	3 (2; 4)	0.17	1.16 (1.04–1.23)
Coated floor	175	167 (95.4)	-	278	278 (100)	-	<0.01	0.37 (0.33–0.42)
Electric light at home	174	166 (95.4)	-	278	266 (95.7)	-	0.89	0.94 (0.37–2.34)
Home with flush toilet	174	146 (83.9)	-	278	275 (98.9)	-	<0.01	0.06 (0.02–0.19)
Sanitary sewage	173	107 (61.8)		278	240 (86.3)		<0.01	0.26 (0.16–0.41)

Not statistically significant: p-value>0.05.

a Years of study.

b Minimum salary in December 2006 for the cases and December 2010 for the control group.

c Compared with wood house. Three mothers in the control group lived in an apartment building.

**Figure 2:**
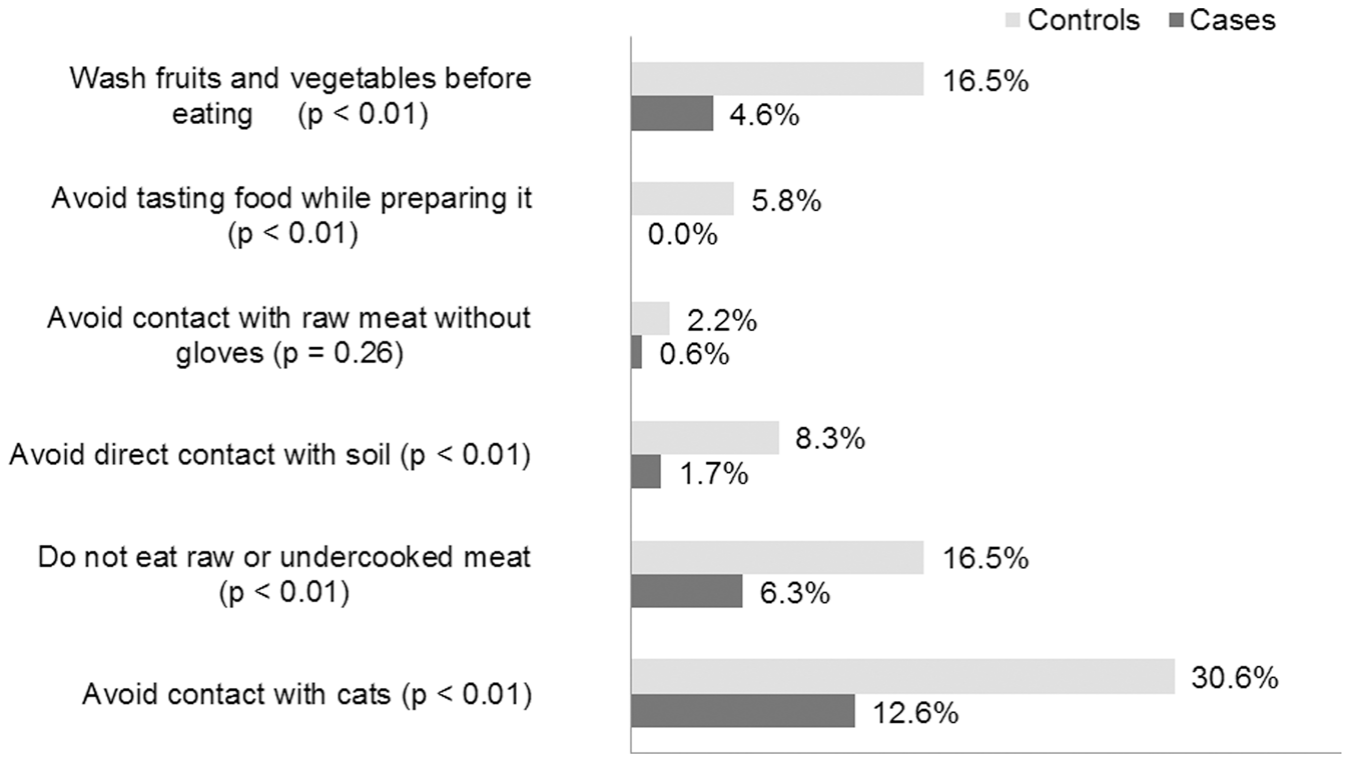
Bar diagram based on the proportion of mothers in both case (175) and control (278) groups who claimed to have knowledge of how to prevent toxoplasmosis.

As shown in Table 2, cases reported fewer prenatal care appointments than controls (p<0.001), but they did not differ significantly in relation to previous guidance about toxoplasmosis prevention (Table 2). However, the number of mothers reporting previous knowledge of the forms of transmission was significantly higher in the control group (Figure 2).

**Table 2:** Univariate analysis of the characteristics of prenatal care provided to the mothers of children with congenital toxoplasmosis and their controls identified in the scope of the newborn screen program of the State of Minas Gerais.

Variables	Cases (n = 175)			Controls (n = 278)			p-value	OR (CI 95%)
	n	Frequency (%)	Median (P25;P75)	n	Frequency (%)	Median (P25;P75)		
At least one prenatal care appointment	175	174 (99.4)		278	275 (98.9)		NS	1.90 (0.20–18.4)
Number of appointments	171		6 (5; 8)	266		8 (6; 10)	<0.01	0.76 (0.69–0.83)
Prenatal care paid with own resources	172	20 (11.6)		272	79 (29.0)		<0.01	0.32 (0.19–0.55)
Inadequate prophylaxis against tetanus	170	9 (5.3)		273	31 (11.4)		0.03	0.44 (0.20–0.94)
Obstetrical ultrasound	174	157 (90.2)		278	275 (98.9)		<0.01	0.10 (0.03–0.35)
Anti-HIV test a	166	128 (77.1)		93	93 (100.0)		NA	NA
VDRL test a	165	156 (94.5)		97	97 (100.0)		NA	NA
HBsAg test a	147	66 (44.9)		89	84 (94.4)		NA	NA
Serology for toxoplasmosis a	168	103 (61.3)		105	94 (89.5)		NA	NA
Prophylactic guidance	174	30 (17.2)		275	45 (16.4)		0.81	1.06 (0.64–1.77)
Guidance at the first appointment b	28	14 (50)		42	17 (40.5)		0.43	1.47 (0.56–3.85)
Guidance to avoid contact with cats	174	28 (16.1)		274	41 (15.0)		0.75	1.09 (0.65–1.84)
Guidance to avoid direct contact with soil	174	5 (2.9)		274	14 (5.1)		0.25	0.55 (0.19–1.55)
Guidance not to consume undercooked meat	174	16 (9.2)		274	29 (10.6)		0.63	0.86 (0.45–1.63)
Guidance to wash fruits and vegetables before eating	174	14 (8.0)		274	32 (11.7)		0.22	0.66 (0.34–1.28)
Guidance to avoid contact with raw meat without gloves	174	1 (0.6)		274	6 (2.2)		0.26	0.26 (0.03–2.16)
Guidance to avoid tasting food while preparing it	174	1 (0.6)		274	7 (2.6)		0.16	0.18 (0.02–1.57)

NA: Not Applicable.

NS: Not statistically significant at p-value>0.05.

a Information unavailable for most controls due to lack of the mother and child health care report.

b Among the women who received any information about preventing toxoplasmosis during pregnancy.

The univariate analysis of the mothers' lifestyle and environmental factors favorable to *T. gondii* transmission pointed to the association of the infection with risk factors related to the three infectious forms of the parasite (Table 3).

**Table 3:** Univariate analysis of the lifestyle and environmental factors favorable to the *T. gondii* transmission among mothers of children with congenital toxoplasmosis and their controls identified in the scope of the newborn screen program of the State of Minas Gerais

Variables	Cases (n = 175)		Controls (n = 278)		*p*-value	OR (CI 95%)
	n	Frequency (%)	n	Frequency (%)		
Owning cats during pregnancy	175	61 (34.9)	278	40 (14.4)	<0.01	3.18 (2.02–5.03)
Cats walking around outside the residence a	61	56 (91.8)	40	32 (80.0)	0.08	2.80 (0.84–9.28)
Report of contact with the cat a	61	24 (39.3)	40	20 (50.0)	0.29	0.65 (0.29–1.45)
Handle cats' feces without glove a	61	9 (14.8)	40	6 (15.0)	0.97	0.98 (0.32–3.00)
Visiting homes where cats live	171	120 (70.2)	273	111 (40.7)	<0.01	3.43 (2.29–5.16)
Owning dogs during pregnancy	175	101 (57.7)	278	148 (53.2)	0.35	1.20 (0.82–1.76)
Eating raw or undercooked meat	171	68 (39.8)	278	64 (23.0)	<0.01	2.21 (1.46–3.34)
Frequency higher than once a week a	68	17 (25)	64	13 (20.3)	0.52	1.31 (0.58–2.97)
Beef a	67	63 (94)	64	53 (82.8)	0.04	3.27 (0.98–10.9)
Pork a	67	26 (38.8)	64	31 (48.4)	0.27	0.67 (0.34–1.35)
Chicken a	67	10 (14.9)	64	4 (6.3)	0.11	2.63 (0.78–8.87)
Fish a	68	3 (4.4)	64	4 (6.3)	0.64	0.69 (0.15–3.22)
Eating fresh meat (not previously frozen)	175	151 (86.3)	278	177 (63.7)	<0.01	3.59 (2.19–5.89)
Drinking treated or boiled water	172	123 (71.5)	278	268 (96.4)	<0.01	0.09 (0.05–0.19)
Drinking raw milk (not boiling it)	174	79 (45.4)	278	98 (35.3)	0.03	1.53 (1.04–2.25)
Frequency higher than once a week a	78	48 (61.5)	98	69 (70.4)	0.22	0.67 (0.36–1.26)
Eating egg with soft yolk	174	63 (36.2)	278	80 (28.8)	0.10	1.40 (0.94–2.10)
Eating raw vegetables away from home	174	114 (65.5)	278	176 (63.3)	0.63	1.10 (0.74–1.64)
Eating away from home during pregnancy	175	152 (86.9)	278	252 (90.6)	0.21	0.68 (0.38–1.24)
Habit of tasting the condiments while cooking a	167	128 (76.6)	272	206 (75.7)	0.83	1.05 (0.67–1.65)
Habit of washing the hands after cooking b	168	135 (80.4)	272	221 (81.3)	0.82	0.94 (0.58–1.54)
Habit of washing the hands before eating	174	129 (74.1)	278	228 (82.0)	0.05	0.63 (0.40–0.99)
Contact with soil during pregnancy	175	76 (43.4)	278	57 (20.5)	<0.01	2.98 (1.96–4.52)
Frequency higher than once a week a	73	33 (45.2)	57	20 (35.1)	0.24	1.53 (0.75–3.11)
Contact with soil without gloves a	75	74 (98.7)	57	53 (93.0)	0.16	5.58 (0.61–51.4)
Livestock	175	81 (46.3)	277	62 (22.4)	<0.01	2.99 (1.98–4.50)
Pigs a	81	30 (37)	62	9 (14.5)	<0.01	3.46 (1.50–8.01)
Cattle a	81	13 (16)	62	10 (16.1)	0.99	0.99 (0.40–2.45)
Rabbits a	81	4 (4.9)	62	4 (6.5)	0.73	0.75 (0.18–3.14)
Birds a	81	73 (90.1)	62	58 (93.5)	0.46	0.63 (0.18–2.19)
Cats in the neighborhood	174	139 (79.9)	276	167 (60.5)	<0.01	2.59 (1.67–4.03)
Reports of cockroach at home	173	114 (65.9)	276	155 (56.2)	0.04	1.51 (1.02–2.24)

NA: Not Applicable.

NS: Not statistically significant at p-value>0.05.

a Among mothers that reported the risk factor.

b Among the mothers that cooked during pregnancy.

In the multivariate model, higher likelihood of congenital toxoplasmosis remained associated with direct contact with the soil, owning and/or visiting homes where cats live, existence of cats in the neighborhood, and consumption of meat that had not been previously frozen. The variables associated with lower likelihood of the disease were: older age of the mother, higher level of education, home with flush toilet, and access to potable water (Table 4).

**Table 4:** Multivariate analysis of the risk factors associated with congenital toxoplasmosis in the State of Minas Gerais.

Variables	Total a		Urban/peri- urban b		Rural c	
	OR (95% CI)	*p* value	OR (95% CI)	*p* value	OR (95% CI)	*p* value
Home with flush toilet	0.18 (0.04–0.78)	0.02	NA	NS	0.06 (0.00–1.01)	0.05
Drinking treated or boiled water	0.21 (0.08–0.51)	<0.01	NA	NS	0.03 (0.00–0.20)	<0.01
Mother's years of education at school d	0.85 (0.78–0.92)	<0.01	0.83 (0.76–0.91)	<0.01	NA	NS
Mother age at delivery d	0.89 (0.85–0.93)	<0.01	0.89 (0.85–0.93)	<0.01	0.88 (0.78–0.98)	0.02
Not a cat owner **and** not visiting homes where cats live (reference)	NA	NS	NA	NS	NA	NS
Owning cats **or** visiting homes where cats live	1.90 (1.09–3.31)	0.02	1.92 (1.08–3.42)	0.03	NA	NS
Owning cats **and** visiting homes where cats live	2.61 (1.19–5.73)	0.02	2.88 (1.21–6.86)	0.02	NA	NS
Cats in the neighborhood	2.27 (1.27–4.06)	0.01	2.10 (1.14–3.85)	0.02	5.77 (1.08–30.9)	0.04
Contact with soil during pregnancy	2.29 (1.32–3.96)	<0.01	2.31 (1.26–4.22)	0.01	NA	NS
Eating fresh meat (not previously frozen)	3.97 (2.17–7.25)	<0.01	4.66 (2.37–9.15)	<0.01	NA	NS

NA: Not Applicable. NS: Not statistically significant at p-value>0.05.

a Cases = 166/controls = 270. Adjusted model – Hosmer & Lemeshow's statistics (*p* value = 0.49).

b Cases = 109/Controls = 250. Adjusted model – Hosmer & Lemeshow's statistics (*p* value = 0.46).

c Cases = 56/Controls = 20. Adjusted model – Hosmer & Lemeshow's statistics (*p* value = 0.99).

d The higher the variable value, the lower the probability of congenital toxoplasmosis.

After stratification according to home in rural or urban/peri-urban area, only two variables remained associated with congenital toxoplasmosis irrespective of stratum: age of the mother and cats in the neighborhood. Home with flush toilet and consumption of treated water were protective against the disease only in the rural stratum (Table 4).

## Discussion

The population-based study reported herein identified important risk factors involved in the epidemiology of congenital toxoplasmosis in the State of Minas Gerais which is the second largest Brazilian State based on number of inhabitants, and its territorial extension is larger than that of France. Among the variables that remained independently associated with the infection, three were related to demographic and socioeconomic characteristics of the mothers, and five were related to the mothers' habits and environmental conditions favorable to the exposure to the parasite.

Several studies carried out in other regions of Brazil have reported significant association of toxoplasmosis seropositivity with poor financial situation, low level of education, and poor house and sanitation conditions [11]–[16]. Confirming reports in the literature, this study shows that congenital toxoplasmosis in the State of Minas Gerais is associated with poor socioeconomic markers both in the univariate analysis (i.e., association with family income lower than one minimum salary) and in the final model (i.e., worst maternal level of education and home without flush toilet).

The association between toxoplasmosis and level of education points to the importance of investing in education as an important strategy for health care promotion. A recent study involving pregnant women at public health care centers in two municipalities in the State of Paraná (Palotina and Jesuítas), also in Brazil, found that the only risk factor significantly associated with the toxoplasmosis prevalence was low level of education [16].

The present study showed that, in Minas Gerais, younger mothers, including adolescents, are significantly more affected by the disease, even after adjustment in the multivariate model. This association has also been found in another Brazilian study carried out in the Municipality of Goiânia, located in the midwest of the country [17]. In addition, a study in the capital of the State of Ceará, in northeastern Brazil, found higher proportion (84%) of risky behaviors for toxoplasmosis infection among 307 pregnant adolescents [18]. These finding and the knowledge that the proportion of women susceptible to toxoplasmosis is higher among younger groups point to the high risk of acquiring the infection during pregnancy in places of high prevalence [19]. Therefore, the present study reinforces the need of prioritizing young women, particularly adolescents, in the educational policies aimed at preventing the disease.

The study also found that prenatal care might fail to guide pregnant women on how to prevent toxoplasmosis. This was true for both cases and controls, irrespective of the higher number of prenatal care appointments in the group of controls. Although quality of prenatal care was not part of this study, this finding suggests that most practitioners have neglected a great opportunity to prevent the disease.

Regarding the possible sources of infection, it is well known that the importance of each varies regionally according to climate, cultural differences of hygiene and dietary habits, as well as farming practices. In Europe, the high prevalence of toxoplasmosis has been associated especially with the consumption of raw or undercooked meat infected with cysts, while in Central America and other developing countries the main factor seems to be environmental exposure to oocysts [20]. Our study found risk factors particularly related to both sources, with predominance of the ones related to the ingestion of oocysts. This is in line with previous studies reported in the Brazilian literature [13], [21], [22].

The consumption of undercooked meat was significantly associated with the infection in the univariate analysis, but did not remain as a strong variable in comparison to other risk factors included in the multivariate model.

The transmission of the parasite through consumption of infected meat is conditioned to the viability of the tissue cysts. Cooking food to ≥67°C for at least 15 minutes is the safest way to eliminate the cysts, but freezing it to under −12°C overnight is also helpful, as most cysts die at cold temperatures [23]. In France, a case-control (1∶1) study of 150 pregnant women found in the univariate analysis that consuming meat that had never been frozen was a risk factor for seroconversion [6]. Bearing this in mind, an interesting finding of the present study was the association of congenital toxoplasmosis with consumption of meat that had not been previously frozen.

Another important risk factor that remained associated with congenital toxoplasmosis in the final model was drinking untreated and unboiled water. Several studies in other regions in Brazil have also reported such an association [11], [13], [21]. Water has been reported as the main risk factor associated with *T. gondii* seropositivity in the population groups at mid or low socioeconomic levels living in Campos dos Goytacazes, a municipality in the State of Rio de Janeiro [11].

As for water, oocysts in soil can also contaminate fruits and vegetables. In Poland, a study, employing molecular technique, detected *T. gondii* oocysts in approximately 10% (21/216) of the sampled fruits and vegetables from groceries and home gardens.[24] Several authors have mentioned the consumption of raw vegetables as a risk factor for toxoplasmosis [12], [13]. This association should not be neglected irrespective of not having been found in our study.

Some authors have reported contact with cats as a risk factor for toxoplasmosis [12], [21], but others have not found this association [14], [25]. One of the possible explanations for the diverging results in the literature may be the fact that the oocysts must mature in the soil for at least one day before becoming infectious. Therefore, the transmission can be avoided when the owners adopt simple measures such as removing the cats' feces daily and cleaning the litter box with boiling water [1]. In the State of Minas Gerais, all variables related with contact with cats – either owning or visiting places with cats – were associated with congenital toxoplasmosis.

Another relevant aspect in our study was the association with the existence of cats in the neighborhood. Considering that the presence of cats in the neighborhood also implies the potential contamination of soil with feces containing oocysts of *T. gondii*, this variable may indeed serve as an important environmental marker of the presence of the parasite.

Toxoplasmosis is an important epidemiologic problem, especially in rural areas, where the parasite has several opportunities to spread [26]. In the State of Rio Grande do Sul, a study with 2,096 pregnant women living 29 municipalities found a high prevalence of *T. gondii* infection in the rural area [21]. The sample of the present study also contained a significantly higher proportion of women living in rural areas among the mothers of infected children. However, this parameter could not be reliably assessed, as a high percentage of controls living in rural areas did not show up for the scheduled interviews.

For this reason, we opted to stratify the sample according to the domicile region. The analysis of the risk factors that remained statistically significant in the final model allowed better understanding of the transmission dynamics of the disease in each of these environments.

Drinking untreated and unboiled water and living in a residence without a flush toilet were risk factors only in the group of mothers living in rural areas. Studies in Poland have stressed the importance of water as a mean of parasite dissemination in rural areas with inadequate hygiene conditions [26], [27].

Another variable that remained statistically significant in the rural stratum was the presence of cats in the neighborhood. This constitutes a substantial problem in this population given the higher survival rates of the oocysts in non-urbanized, unpaved areas as well as the probability of oocyst dispersion by rainwater [28].

In the urban and peri-urban strata, the variables that lost significance were drinking untreated and unboiled water and living in a home without flush toilet. This finding probably reflects the more homogeneous access of the urban population to municipal water supply and sanitary infrastructure in general.

This study carries some limitations, which are due not only to the case-control approach, but also to a few choices made. The most relevant limitation corresponds to the process of selecting and classifying the control group.

Because of operational and financial constraints, we could not seek the controls in the same calendar year in which the cases were born. Although no state or national policy for control of congenital toxoplasmosis changed in study period [29], the overall socioeconomic conditions of the Brazilian population improved in the period. Therefore, the association of congenital toxoplasmosis with younger age of the mother and lower level education may have been overestimated in our cross-analysis of cases and controls. However, even comparing the cases with the general population in the State of Minas Gerais in 2007 (available at http://tabnet.datasus.gov.br/cgi/deftohtm.exe?sinasc/cnv/nvMG.def), one can observe a significantly higher proportion of adolescent mothers and less educated mothers (less than seven years at school) among the cases (respectively 31.4% and 56.0%) than among the general population (respectively 18.7% and 39.6%). Additionally, we compared the proportion of population in Minas Gerais State served by sewage and water supply in both research periods (2007 and 2011) using available information on proxies for economic changes. They were respectively 79.4% and 79.7% for the former and 85.9% and 85.2% for the latter (http://tabnet.datasus.gov.br/cgi/idb2012/matriz.htm#socio). Although resulting in significant *p* values (<0.05), probably because of the sample size, we can see that those changes were rather small.

Sampling of controls followed some criteria as a means of preserving sample representativeness, and no significant difference was found across the answers provided by random and selected controls. Another limitation was that the controls children were older than cases by the time of interview. In order to investigate a potential information bias, a sub-analysis was carried out including only the mothers (case and controls) of the children aged between 50 and 110 days. That interval was chosen considering the age median of group of controls and cases. This sub-analysis showed no overall changes in the main associations found in this study.

Finally, as the children in the control group were not serologically screened for toxoplasmosis, a few cases might have been inadvertently included in the control group, reducing the association estimates. This is a remote possibility, considering the prevalence of 13 cases of congenital toxoplasmosis for every 10 000 liveborn infants in Minas Gerais. If this classification error did take place, however, it was not sufficient to hamper the identification of associations in our study.

Case-control studies are classically subject to recall bias, as the mothers of the affected children may be more likely than the others to remember of factors related to their exposure [10]. To investigate this bias, risk factors among the cases were analyzed in the light of the children's clinical manifestation, or the mothers' perception of the severity of their situation (data not shown). This analysis pointed to no significant difference, suggesting low likelihood of this type of bias.

In conclusion, this population-based investigation adds valuable contributions to the understanding of risk factors for congenital toxoplasmosis in Minas Gerais State. The differences between populations living in rural and urban areas regarding the main risk factors for toxoplasmosis point to the need of considering regional specificities in planning strategies to control congenital toxoplasmosis. In the urban area, most affected mothers were younger and had lower educational attainment, pointing to the potential value of focusing on primary preventing measures among these subgroups. The association between consumption of fresh meat that had not been frozen serves to warn surveillance authorities to implement careful inspection of the meat available for human consumption. In the rural area, where risk factors related to the ingestion of oocysts and poor sanitation predominated, priority should be given to the public measures aimed at improving sanitation.
